# Historical ecology reveals landscape transformation coincident with cultural development in central Italy since the Roman Period

**DOI:** 10.1038/s41598-018-20286-4

**Published:** 2018-02-01

**Authors:** Scott A. Mensing, Edward M. Schoolman, Irene Tunno, Paula J. Noble, Leonardo Sagnotti, Fabio Florindo, Gianluca Piovesan

**Affiliations:** 1 Department of Geography, University of Nevada, Reno, Nevada 89557 USA; 2 Department of History, University of Nevada, Reno, Nevada 89557 USA; 3 Center for Accelerator Mass Spectrometry, Lawrence Livermore National Laboratory, Livermore, CA 94550 USA; 4 Department of Geological Sciences and Engineering, University of Nevada, Reno, Nevada 89557 USA; 5 Istituto Nazionale di Geofisica e Vulcanologia, Rome, Italy; 6 Dendrology Lab, DAFNE Universita degli Studi della Tuscia, Viterbo, 01100 Italy

## Abstract

Knowledge of the direct role humans have had in changing the landscape requires the perspective of historical and archaeological sources, as well as climatic and ecologic processes, when interpreting paleoecological records. People directly impact land at the local scale and land use decisions are strongly influenced by local sociopolitical priorities that change through time. A complete picture of the potential drivers of past environmental change must include a detailed and integrated analysis of evolving sociopolitical priorities, climatic change and ecological processes. However, there are surprisingly few localities that possess high-quality historical, archeological and high-resolution paleoecologic datasets. We present a high resolution 2700-year pollen record from central Italy and interpret it in relation to archival documents and archaeological data to reconstruct the relationship between changing sociopolitical conditions, and their effect on the landscape. We found that: (1) abrupt environmental change was more closely linked to sociopolitical and demographic transformation than climate change; (2) landscape changes reflected the new sociopolitical priorities and persisted until the sociopolitical conditions shifted; (3) reorganization of new plant communities was very rapid, on the order of decades not centuries; and (4) legacies of forest management adopted by earlier societies continue to influence ecosystem services today.

## Introduction

The impact of past societies on changing global biodiversity is well established and the extent and direction of human-induced environmental change has significant implications for conservation strategies and maintenance of ecosystem services^1^. Understanding the direct role humans have had in shaping the environment requires the perspective of historical and archaeological sources, as well as climatic and ecologic processes, in interpreting paleoecological records^2^. While the sum of human activity has produced effects measurable at the global scale^3,4^, humans act at the local scale, and understanding the direct link between human activity and environmental change is critical in distinguishing between human and climatic drivers of ecologic change. There are still insufficient empirical studies that combine high-resolution paleoecological records with historical documents and archaeological data to illuminate how changing sociopolitical priorities influenced local land use^5^, to some extent because there are few places with high-quality datasets that allow the comparison of all three sources in a single locality^6–9^.

This paper builds upon previous paleoecologic reconstructions from the Rieti Basin in central Italy^10,11^ by comparing climatic and ecologic change with detailed historical texts from the Roman Period to the present to better inform our understanding of the causal relationship for abrupt environmental change, and document the extent to which modern ecosystem services are a result of human land use practices. We found that: (1) abrupt environmental change was more closely linked to political/regime change than climate change; (2) landscape changes reflected the new sociopolitical priorities introduced by each regime and generally persisted until the sociopolitical conditions changed; (3) reorganization of new plant communities was very rapid, on the order of decades and not centuries; (4) legacies of forest management adopted by earlier societies continue to influence ecosystem services today.

## The Rieti Basin

The Rieti Basin is an intermontane depression in the central Apennines 70 km north of Rome and connected to that city by the ancient Via Salaria^12^. The basin, located at the northeastern edge of Lazio, had been in the historical age under the control of the Romans, Ostrogoths, Lombards, Carolingians, and part of the Duchy of Spoleto, Kingdom of Italy, Holy Roman Empire, and Papal states. Historical documents related that in the early Middle Ages (750 CE–900 CE), parts of this territory had been under the local control of the Monastery of Farfa, and later under the Diocese of Rieti itself, while extensive archaeological surveys have revealed a continuous occupational history from the third century BC^11,13^. The basin held a large shallow lake (*Lacus Velinus*) between ~6000 and 3000 yr BP^14^. Classical Latin histories describe the Romans cutting a channel through the calcareous tufa sill at the point of discharge of the Velino River over Marmore falls ~270 BCE^12^, partially draining the basin wetlands for reclamation of pasture. Continued hydrologic control has played a major role in environmental changes in the valley^15^. Historical maps portray substantial changes in the size and shape of lakes in the basin, their proximity to the Velino River, and the extent of wetlands through time. Today, four remnant lakes persist; Lago Lungo, Ripasottile, Ventina and Piediluco. Our site, Lago Lungo, (369 a.m.s.l.), presently has a maximum depth of 4.5 m with a surface area of 0.41 km^2^ ^16^. Modern vegetation is dominated by maize and cereal cultivation in the basin and heavily managed coppice, dominated by oak (*Quercus pubescens* and *Q. cerris*) and hop hornbeam (*Ostrya carpinifolia*) forest on the surrounding slopes. Rieti is a seismically active extensional basin within the Apennine thrust system and is partially filled with Upper Pliocene-Holocene continental and marine sediments^17,18^. Sedimentation rates are very high, locally averaging 3–12 mm yr^−1^ ^10^ and attributed to the high levels of catchment erosion due to agriculture^19^ and forest coppice^20^.

## Results

### Chronology

**Figure 1:**
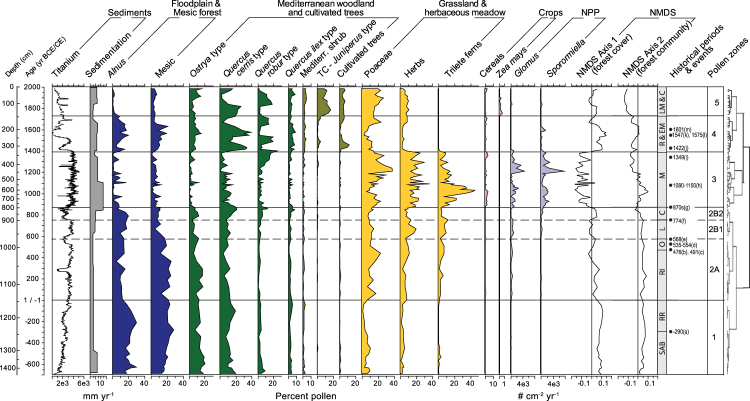
Selected sedimentary data, pollen types, non-pollen palynomorphs and non-metric multidimensional scaling (NMDS) scores from pollen data for Lago Lungo plotted against core depth and age (Common Era). Titanium obtained using XRF is reported in kilacounts per second. Summary pollen groups (e.g. Mesic) were aggregated on the basis of the NMDS ordination: Mesic taxa include: *Ostrya, Fagus, Carpinus, Acer, Ulmus, Fraxinus excelsior, Betula*; Mediterranean shrubs include: *Fraxinus ornus, Pistacia*, and *Myrtus*; Cultivated trees include *Olea, Castanea* and *Juglans*; Herbs include: Apiaceae, *Plantago*, Amaranthaceae, *Trifolium, Galium, Polygonum, Salvia*, Caryophyllaceae, Asteraceae, and Cichorieae; Poaceae includes Cyperaceae. The Q-mode cluster of samples—constrained by stratigraphic order—recognizes 5 main groups of samples, with group 2 further divided into three clusters. Historical periods associated with pollen zones are SAB—Sabini; RR—Roman Republican; RI—Roman Imperial; O—Ostragoth; L—Lombard; C—Carolingian; M—Medieval; R&EM—Renaissance and Early Modern; LM&C—Late Modern and Contemporary. Historical dates noted in the figure are (**a**) conquest of *Manius Curius Dentatus* and construction of *Cava Curiata*; (**b**) Last Roman emperor deposed; (**c**) Beginning Ostragoth occupation; (**d**) Gothic wars; (**e**) Lombard conquest; (**f**) Charlemagne conquest; (**g**) Land charter phase; (**h**) Hill-town charter phase; (**i**) Black Plague; (**j**–**m**) Canal construction phase.

Due to significant challenges with ^14^C we developed a chronology using paleomagnetic secular variation (PSV) fit to the well-dated archaeomagnetic PSV model for Europe (ref.^21^; see Methods). The error on the paleomagnetic age model varies from a minimum 2σ error of 49 yr (1320 CE) in the upper part of the core and a maximum of 200 yr (300 BCE) in the lower part of the core, with an average of 109 yr^10^. The date for the base of the core is 700 BCE. Based on the above-mentioned sedimentation rates, the time between pollen samples ranges from 10 to 60 years (average = 30 yrs). Boundaries between pollen zones identified through cluster analysis (ref.^22^, Fig. 1) and representing significant shifts in vegetation, coincide with historic periods developed from previous archaeological studies of well-established ceramic sequences in the Rieti Basin^12^. Zone 1, from 700 BCE to 1 CE, corresponds to the Archaic, pre-Roman (Sabini) and Roman Republican periods; Zone 2, divided into two subzones, 2 A from 1 CE to 600 CE, corresponds to the Roman Imperial period through Late Antique period, and Zone 2B from 600 to 875 CE, corresponds to the Early Medieval period of Lombard occupation (2B1, 600–750 CE) and the Carolingian conquest (2B2, 750–875 CE); Zone 3, from 875 to 1400 CE, corresponds to the end of the early Medieval through Late Medieval period; Zone 4, from 1400 to 1750 CE, corresponds to the Renaissance and Modern period; and Zone 5, from1750 CE to present, corresponds to the late Modern through Contemporary periods. The uncertainties in our age model do not allow us to claim perfect synchronicity between the pollen record and the historical record, but the very high sedimentation rates and close sampling interval do allow us to confidently identify the rapidity of ecologic change from one period to the next as well as the duration of each landscape period. The interpretation of historical texts suggests changes in land use consistent with what we see in the pollen record, lending confidence to our interpretation that the periods defined in the pollen record represent the historical periods described by historians and archaeologists.

### Vegetation structure

**Figure 2:**
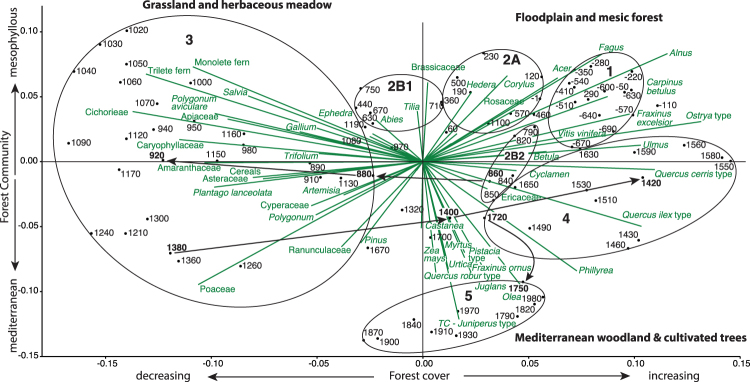
Non-metric multidimensional scaling (NMDS) based on Bray-Curtis similarity index (stress = 0.14; R^2^ of the axis 1:0.64 and of axis 2:0.19) of the plant taxa (green text) from Lago Lungo. The length of the vectors (green lines) are arbitrarily scaled to make a readable biplot; only their directions and relative lengths should be considered. Black filled circles represent the location in biplot space of pollen strata; associated numbers represent ages (year BCE/CE) for each pollen strata. Suggested pollen-derived vegetation communities are given for each quadrant. Numbered ellipses contain the majority of samples from each pollen zone (1–5) from Fig. 1 to represent the time series data in ecologic space. Arrows reference specific rapid transitions from a forested landscape to predominantly grassland, a return to forest, and shift to modern Mediterranean woodland.

The discrete changes in vegetation structure identified by cluster analysis was confirmed by NMDS ordination (ref.^23^, Fig. 2). Each temporal period (Zones 1–5) occupies nearly entirely non-overlapping ecologic space suggesting that during each successive historic period a new landscape was created. The primary axis in the NMDS ordination plot explains 64% of the variance, and is interpreted as a measure of forest cover (r = 0.98 p < 0.001; between axis 1 and % tree pollen). The secondary axis explains 19% of the variance and we interpret this as a measure of change in forest community between more mesic taxa and more Mediterranean taxa. Vectors indicating the relationships between plant taxa are consistent with established knowledge of forest ecology^24^. Three distinct communities were identified; (1) floodplain and mesic forest represented by alder (*Alnus glutinosa*), beech (*Fagus sylvatica*), hop hornbeam (*Ostrya carpinifolia*), ash (*Fraxinus excelsior*), hornbeam (*Carpinus betulus*), elm (*Ulmus* spp.), lime (*Tilia* spp.) and maple (*Acer* spp.); (2) Mediterranean woodland and cultivated trees represented by oaks: Turkey oak (*Quercus* subg*. cerris)* in more mesic environments, downy oak *(Quercus robur* type) in more arid environments, and evergreen oaks (*Quercus ilex)*; pioneering Mediterranean woody taxa represented by juniper and cypress (Cupressaceae), ash (*Fraxinus ornus*), myrtle (*Myrtus communis*), *Pistacia* (spp.) and *Phyllyrea variabilis*; cultivated tree taxa represented by olive (*Olea europa*), walnut (*Juglans regia*), and chestnut (*Castanea sativa*), and field crops including hemp (*Cannabis stativa* var*. vulgaris*) and corn (*Zea mays*); and (3) grassland and herbaceous meadow represented by grass (Poaceae spp.) and a wide range of disturbance taxa including ferns (Trilete), chicory (Cichorieae), amaranth (Amaranthaceae), plantain (*Plantago* spp.), Apiaceae, sunflower (Asteraceae), *Polygonum* spp., *Salvia* spp., *Gallium* spp., *Trifolium* spp. and carrot (Caryophyllaceae).

### Climate proxies

**Figure 3:**
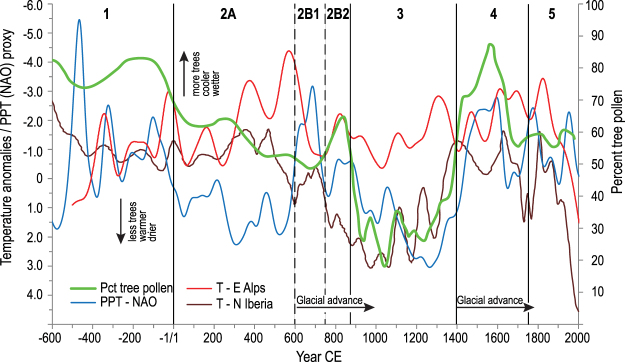
Comparison of trends in percentage tree pollen (Floodplain, Mesic and Mediterranean woodland as defined in Fig. 2), temperature (T^27,30^;) and North Atlantic Oscillation proxies (NAO^25^;). T^27^ and NAO^25^ values were rescaled dividing respectively by a factor of 3 and 4. All climatic proxy data were multiplied by a factor of 10. Glacial advances are for the Calderone Glacier^31,32^. Numbered divisions are the 5 pollen zones described in Fig. 1.

Forest abundance (percent tree pollen) correlates with precipitation with a few notable exceptions, for most of the record (Fig. 3); percent tree pollen typically increases during periods of negative North Atlantic Oscillation (NAO; increased precipitation, ref.^25^) and decreases during periods of positive NAO (decreased precipitation) in agreement with variation in forest productivity^26^. This relationship is weakest during the Lombard period (600–750 CE) and the contemporary industrial period when percent tree pollen declines despite the NAO remaining negative. Regional temperature proxies are generally coherent, but the Alps record^27^ shows significant cooling between 300 and 600 CE. The coldest phase of this period, from 536–660 CE, has recently been referred to as the Late Antique Little Ice Age (LALIA, ref.^28^), and falls within the period between 410 and 775 CE that has previously been called the Dark Ages Cold Period (DACP, ref.^29^). The LALIA is not as strongly expressed in the Northern Iberian temperature signal^30^. The Calderone Glacier^31,32^ did not advance before 600 CE, suggesting either that central Italy did not experience such cold temperatures, or there was insufficient moisture for glacier growth. There is an inverse relationship between temperature and percent tree pollen, primarily seen during the Medieval period (900–1400 CE) with less tree pollen during warmer periods and more during cold periods, especially at the beginning of the Little Ice Age (LIA).

## Discussion

The pre-Roman Rieti landscape was dominated by a diverse mesic deciduous forest on the uplands and a wetland forest in the basin (Figs 1 and 2; Zone 1). Direct modification of the environment began shortly after conquest by *Manius Curius Dentatus* in 290 BCE, and subsequent construction of a channel (*Cava Curiata*) cut through the calcareous tufa at *Marmore* to lower the water level and reclaim portions of the basin^12^. We see evidence for drainage through the loss of *Alnus* (a wetland obligate), but no significant impacts on the other woodland prior to the Imperial Roman period. During the Imperial Period (Zone 2A), although there is evidence of intensification of land use in portions of the western Mediterranean^33^, studies from Italy show much less evidence for widespread pressure on forest resources^34^. In the Rieti basin, settlement was primarily villas concentrated in the basin edges and low hills^13^ and the priority for land use was pasture for high-quality horses and stock according to the contemporary account of Varro, *De re rustica 3.2*. This produced an expansion of pasture, and some loss of mesic forest between 1 and 400 CE, without any dramatic change in forest composition (Figs 1 and 2). In the following century, the capital moved from Rome to Milan then Ravenna, the last western Roman emperor was deposed in 476 CE, and the Ostrogoths began their occupation of Italy in 491 CE. Though temperature reconstructions from the Alps record a cold period corresponding to the Migration Period^27^ characterized in other portions of the Roman Empire as one of political turmoil compounded by exceptional climatic variability and forest expansion^27,28^, historical sources note that during the fifth century following the invasion of Rome, under Ostrogothic rule the Roman system of governance, taxation, and land management, continued in Rieti into the 6^th^ century with remarkably little impact on the environment^35,36^. There is no forest recovery associated with the Ostrogothic conquest and end of Roman rule but rather minor loss of mesic forest, likely associated with greater reliance on local resources as the extensive Roman trade network disintegrated^37^.

The Gothic wars (535–554 CE) are thought to have depopulated central Italy and contemporary medieval writers recount a period of reforestation along with the abandonment of settlements^38^. Following the Lombard conquest (568 CE), Rieti fell under the control of the semi-independent Duchy of Spoleto in 592 CE. Archaeological absence of new settlements during this period further argues for depopulation of the land. Climatic indices (Fig. 3) suggest this was a wetter and cooler period, with initiation of glacial expansion in the Apennines, which should favor afforestation of the floodplain and the spread of mesic species. Despite this, after 600 CE a new more open forest emerges (Zone 2B1) with the loss of particular ‘soft’ hardwoods dominant in the mesic forest, including beech, ash, elm, maple, and hornbeam, that appear to have been targeted for cutting (Fig. 1). While there are few written records specific to Rieti, compared to their Roman predecessors Lombards constructed buildings chiefly in wood, which needed replacement between every 10 and 50 years^39^, and placed a greater value on production of livestock, particularly pigs^36,40–42^. New priorities for wood and meat created a functionally different landscape with less biodiversity. Indicators of erosion (*Glomus*) and livestock (*Sporormiella*) support an interpretation of a more open grazed woodland despite a warmer wetter climate and potentially lower population. This landscape persisted until the late 8^th^ century when northern Italy was once again conquered by a new power, the Frankish dynasty of the Carolingians.

In 774 CE, Charlemagne gained control of the Kingdom of Italy, and the following year the Monastery of Farfa was elevated to the status of an imperial monastery; the next century saw an increase in donations and the region as a whole became more aligned with Rome^43^. The landscape again quickly reorganized with rapid afforestation dominated by oaks (Figs 1 and 2). The temperature reconstructions from northern Iberia and the eastern Alps have opposite signals during this period (Fig. 3, 2B2) but advance of the Calderone Glacier indicates that this was a cool period, consistent with an extended DACP^29^, which should have favored mesic taxa such as beech, ash and elm. Rapid reforestation of oaks may have been a response to the forests being more intensively managed for the raising of pigs, an increasingly important food source^41^.

A substantial ecologic change occurs between 875 and 900 CE. Pollen of floodplain and mesic forest and Mediterranean woodland rapidly decrease and cultivated cereals, grass, ferns and herbaceous taxa increase. During this period, the temperature, precipitation and pollen signals are synchronized (Fig. 3) recording warmer temperatures (Medieval Warm Period), less precipitation and fewer trees (Fig. 3). Forest clearing is common throughout Europe during the Medieval Period^3^, suggesting a link to climate, but the timing and rapidity of forest loss in Rieti is far greater than can be explained by climate alone. Archaeological evidence from much of Europe emphasizes an episodic change in rural land management and agricultural intensification in the mid-9^th^ century along with relatively fluid structures^36,44,45^. In the 870 s there is an intensive production of charters including a large number of 29-year leases highlighting the monastery’s new role in land management^46^. The term *Rosea*, used since the Roman Period to describe a landscape of forest and reclaimed lake-side pasture, no longer appears in historical documents after 825 CE^47^. New community patterns, hilltop fortified settlements termed *incastellamento*^48^ appeared at this time. A warmer climate (Fig. 3) likely allowed the upslope expansion and persistence of these settlements at elevations as high as 1000 m with many hill-town charters first appearing in the Rieti documents between 1080 and 1150 CE^12^. Locally forests were managed on a short 7–14 year clear cut cycle^49^ and a fragmented and degraded forest landscape persisted until the late 14^th^ century.

The conversion from grassland back to forest was equally rapid, between 1380 and 1400 CE (Fig. 2). It is likely that the medieval practice of coppicing and of pollarding trees rather than completely clearing forest had left many stems capable of resprouting, resulting in more rapid forestation. This landscape change coincided with wetter and cooler climate of the Little Ice Age (LIA) (Fig. 3) but the rapidity of forest recovery was likely aided by depopulation following the 1349 CE black plague and its recurrence in 1363, 1374, 1384, and 1400 CE as well as famine following a locust invasion in 1365, that reduced Rieti from 8000 to 4000 people by the end of the 14^th^ century^50^. Further complications arose from three large earthquakes that struck the region on September 9, 1349^51^. Loss of human labor combined with wetter cooler climate constrained efforts to drain the valley, favored tree growth and limited the ability for land clearing and agriculture. New laws were implemented to control the use and maintenance of cultivated trees, particularly chestnut, walnut and olive^52^. Documents report increasing reliance on game over livestock and the return of wolf and bear to the forests is noted^53^. Forest expansion is coincident with a negative phase of the NAO (increased precipitation; Fig. 3). Decreased titanium, a proxy for siliciclastic input in the lake sediments (Fig. 1) provides evidence of decreased hillslope erosion, or overbank flooding from the Velino River. Lake sediment becomes marlier (increased CaCO_3_ content, Supplementary Fig. S1) and is coincident with a short-lived phase of calcareous tufa aggradation in the basin of the Nera River, immediately downstream from *Marmore*^54^. Expansion of *Alnus* during this period supports expansion of a wetland forest in the basin (Fig. 1). Repeated efforts by the community to drain the valley through new canal projects in 1422, 1547, and 1575 CE^15,55^ demonstrate the challenge presented by excess water. In 1601 the application of new hydrologic technology developed during the Renaissance^15,55^ combined with a shift towards a positive NAO and drier climate led to the eventual draining of the basin and conversion of wetland forest into agricultural land.

The contemporary landscape occupies a unique ecologic niche dominated by pioneering shrubs and early successional Mediterranean woody species (Figs 1 and 2). Forest laws from the mid 18^th^ century mandated coppicing on a 9-year rotation and required authorization prior to converting forest into agricultural land^49^. This new coppice system continued to open mesic forests and created an opportunity for Mediterranean pioneer species to invade large portions of the perturbed forest and become important for the first time. Juniper spread in the basin along with sub-Mediterranean early successional species like deciduous oaks (e.g. *Quercus pubescens*) and hop hornbeam. In the valley, the Velino River was channelized and the basin was completely drained to allow for expansion of agriculture and the introduction of new crops (e.g. *Zea mays*).

The trajectory of landscape construction associated with each subsequent period of land management has resulted in a novel landscape that has no resemblance to any previous landscape of the last 2700 years and appears to be ecologically different from the floodplain and mesic forest that predominated prior to the Roman period.

By tracing forest change through multiple polities, each with their own sociopolitical priorities, we are able to reconstruct the process by which humans have created a sequence of new ecologic niches. The result has been a forest that has lost few tree taxa through time (e.g. *Abies*), yet today represents an entirely new forest ecosystem. We found that each political regime created a new landscape with different levels of forest biodiversity and ecosystem functions when compared to the previous regime (Supplementary Fig. S2). New forest types were emplaced rapidly and then persisted through the duration of that regime. Each period shaped a natural heritage that constrained forest dynamics under the subsequent regime, such that the ecosystem today is a legacy of a long series of natural and sociopolitical processes. Most recently, the draining of the basin and conversion of floodplain forest to permanent agriculture and opening of mesic forest combined with very high rates of soil erosion^20^ from short rotation coppicing seems to have produced a large scale aridification in the watershed and expansion of Mediterranean shrubs, which were not an important ecological component at any previous period. The modern landscape carries the signature of each preceding regime and thus, the elements of the modern ecology can be best understood through tracing how each cultural shift, combined with climatic change, produced a legacy that has accumulated to create the present forest composition and structure.

Although the Romans and Ostrogoths impacted the environment^56^, the first persistent legacy impact within the last 2700 years was a reduction of species richness among specific ‘soft’ hardwoods targeted by the Lombards. Archaeologists and historians have suggested that populations during the Lombard period were lower, especially in cities^43^. If this is true, then it suggests that smaller populations that target specific species can have a significant and permanent effect on forests^57,58^. In the subsequent period of forest recovery under the Carolingians, oaks became dominant and although mesic taxa recover, they never return to their prior abundance. Whether oaks predominate because land management focuses on pig production or whether it is due to the secondary succession of oaks following the reduction of floodplain and mesic taxa we cannot determine. What is certain is that during this short period of forest recovery there was no return to the original forest ecosystem.

The most dramatic and lasting impact on forests is during the Medieval period when forest species richness reached a low point (Supplementary Fig. S2). No taxa become locally extinct, but the percent pollen from mesic taxa falls below 10% indicating a profound alteration of the forest landscape. The pattern of reforestation following the period of the Black Plague is similar to sites across Europe^59,60^, but the details from Rieti have implications for modern conservation strategies and maintenance of ecosystem services. In the two centuries between 1400 and 1600 CE, floodplain forest (*Alnus*) returned to the valley and mesic forest expanded on the slopes. In the absence of intensive human impacts, and aided by favorable climate, forest succession was proceeding towards the original floodplain and mesic forest, although with a higher percentage of Mediterranean oaks (*Q. cerris* and *Q. ilex*). A period of two centuries or more for forest recovery is consistent with known forest dynamics^24^ and the longevity of European deciduous trees that have a typical lifespan of 200–300 years^61^. But this trajectory was reversed after1601 by construction of a successful drainage system that eliminated virtually all floodplain forest by 1750 CE and converted the former wetlands into permanent agricultural fields with settlements (Fig. 2). Expansion of invasive juniper coincided with the permanent loss of valley wetlands as well as new forest management laws that promoted short-rotation coppicing in forests and created some of the highest erosion rates in all of Europe^19^. The modern landscape appears to represent an aridification of the watershed linked to the priority for permanent agriculture and settlement in the valley in place of wetlands. Although the modern forest cover (Fig. 1; NMDS axis 1) is equivalent with that of Late Antiquity, and the forests are managed to reduce impacts (grazing, fire and frequent cutting) there is no expansion of mesic forest equivalent to the post-Medieval period (Fig. 1; NMDS axis 2). The paleoecological reconstruction suggests that the mesic forest and floodplain wetland are linked, and restoration of the mesic ecosystems is not likely to be accomplished without restoration of the floodplain ecosystems and a return of a more humid environment within the basin. The longstanding priority of draining the wetlands represents a challenge to the goal of sustainable forest conservation and its associated ecological services.

Efforts to manage biodiversity in the ecosystem today, or to try to restore elements of the former forest ecosystem, must consider that modern niches are the unique consequence of a long and complicated series of sociopolitical decisions that have helped shape the environment, and not just the result of a sequence of ecological and climatological processes. The history of human sociopolitical change is as diverse and geographically complex as is the history of ecological processes and we need to recognize that while the sum total of human activity is widely acknowledged to have contributed to global environmental change, the process of how this happens is likely very different from place to place and detailed analyses of localities can provide important insights into the specific causes of environmental change.

## Materials and Methods

Overlapping sediment cores (LUN09 in 2009 and LUN12-1A, 1B, 2A, and 2B in 2012) were recovered from an anchored floating platform with a modified square-rod Livingstone corer^10^. The 14.4 m composite core was split and imaged with a digital line scanner at ~300 dpi, and magnetic susceptibility measured every 0.5 cm with a Geotek MSCL-XYZ. Magnetic susceptibility and imagery were used to correlate core sections. Natural and artificial magnetizations were measured at room temperature on a narrow-access (45 mm diameter) automated pass through ‘2 G Enterprises’ DC 755 superconducting rock magnetometer (SRM), housed in a Lodestar Magnetics shielded room. Rock magnetic and paleomagnetic properties were measured at 1-cm spacing on u-channel samples collected from 4 distinct and partly overlapping cores (LUN09, LUN12-1A, LUN12-1B and the lower 5.4 m of LUN12-2B).

Development of an age model was challenging. The carbonate bedrock introduced significant old-carbon effects and plant macrofossils were absent from most of the core. Measures of ^137^Cs and ^210^Pb activity and the presence of the introduced crop *Zea mays* pollen at 134 cm depth constrained the age at 134 cm to between 1700 and 1750 CE. AMS ^14^C ages on macrofossils from 159 and 170 cm depth produced dates of 680 and 1175 CE^10^. We developed an age model from fifteen ^14^C AMS dates between the 159 and 1000 cm depths. The majority of macrofossils were found clustered within a one-meter section of core, causing us to suspect they may have been transported to the site during periods of flooding, erosion and possibly redeposition of older sediments. We also constructed a separate PSV based age model^10^ from biostratigraphic markers (*Zea mays*, *Cannabis sativa* var. *vulgaris*) and paleomagnetic trends (declination, inclination and paleointensity) correlated to available paleosecular variation curves and models for Europe^21^. All three paleomagnetic stratigraphic trends (inclination, declination and intensity) through the full length of the core were tied to those predicted by the European PSV model for the last 3 ka^21^ and the chronology was consistent with the ^210^Pb and ^137^Cs data. After extensive comparison reviewed in detail elsewhere^10^, we concluded that the PSV age model produced the most accurate chronology.

Ninety-one samples were analyzed for pollen at ~10–20 cm intervals; eighty-nine pollen types, six non-pollen palynomorphs (NPP) and eleven algae types were identified. Pollen percentages were calculated from the sum of terrestrial pollen, excluding indeterminate grains and *Cannabis* type (which was retted in the lake at certain periods^62^. Accumulation rates (grains cm^−2^ yr^−1^) were calculated by dividing concentration (grains cm^−3^) by the number of years per sample (yr cm^−1^) and normalizing by number of *Lycopodium* (a known sum of exotic spores added to the samples) counted. Selected NPP are presented as accumulation rates. Chronologic zonation was identified using a multi-variate analysis constrained single-link dendrogram created using CONISS in the PolPal plotting program^22^. Data input included the fifty-three terrestrial taxa with at least one strata of >1% of the pollen sum, excluding *Cannabis* type and indeterminate grains. Detailed core description and age model development have been previously published^9^.

Non-metric multidimensional scaling (NMDS), based on Bray-Curtis distances calculated on taxa pollen percentages, was used to summarize the multivariate time matrix in a low (biplot) dimensional space^23^. NMDS can indirectly reveal the presence of gradients to assess ecological community dynamics, and provide support for comparing the timing of abrupt ecological change with historical events. Percentages of tree pollen species, assembled following NMDS results, were compared with precipitation^25^ proxy data (https://www.ncdc.noaa.gov/paleo-search/study/18516) and temperature^27,30^ proxy data (https://www.ncdc.noaa.gov/paleo-search/study/12194; https://www.ncdc.noaa.gov/paleo-search/study/10394), and geochemical and sedimentological parameters^10^. Since existing climatic reconstructions for the Italian peninsula that span the last 3000 years are largely based on pollen^63–65^, we selected two high-resolution temperature reconstructions from the region that each showed good spatial correlation with the Rieti basin, and were based on independent evidence; a δ^13^C speleothem record from northern Iberia^30^ and a tree-ring record from the eastern Alps^27^. We also include reconstructed glacial advances of the Calderone Glacier (Fig. 3) that has low temporal resolution, but provides a local proxy of cooling temperatures^31,32^. Temperature, North Atlantic Oscillation (NAO) indexes, and pollen series were smoothed with a Gaussian filter (weighted moving average using a Gaussian kernel with standard deviation set to 1/5 of the window size^23^.

### Data availability

The datasets generated during and/or analysed during the current study are available in the European Pollen Database repository. http://www.europeanpollendatabase.net/index.php.

## Electronic supplementary material

Supplementary Information
