# Supplementary material for: Historical ecology reveals landscape transformation coincident with cultural development in central Italy since the Roman Period
Source: Sci Rep. 2018 Feb 1;8:2138. doi: 10.1038/s41598-018-20286-4 (PMC5794987; doi:10.1038/s41598-018-20286-4)
Supplement: Supplementary file 1 — Supplementary Information [file 41598_2018_20286_MOESM1_ESM.pdf]

Supplementary Information

"Historical ecology reveals landscape transformation coincident with cultural development in central Italy since the Roman Period"

Mensing S, E Schoolman, I Tunno, P Noble, L Sagnotti, F Florindo and G Piovesan

Supplementary Figures

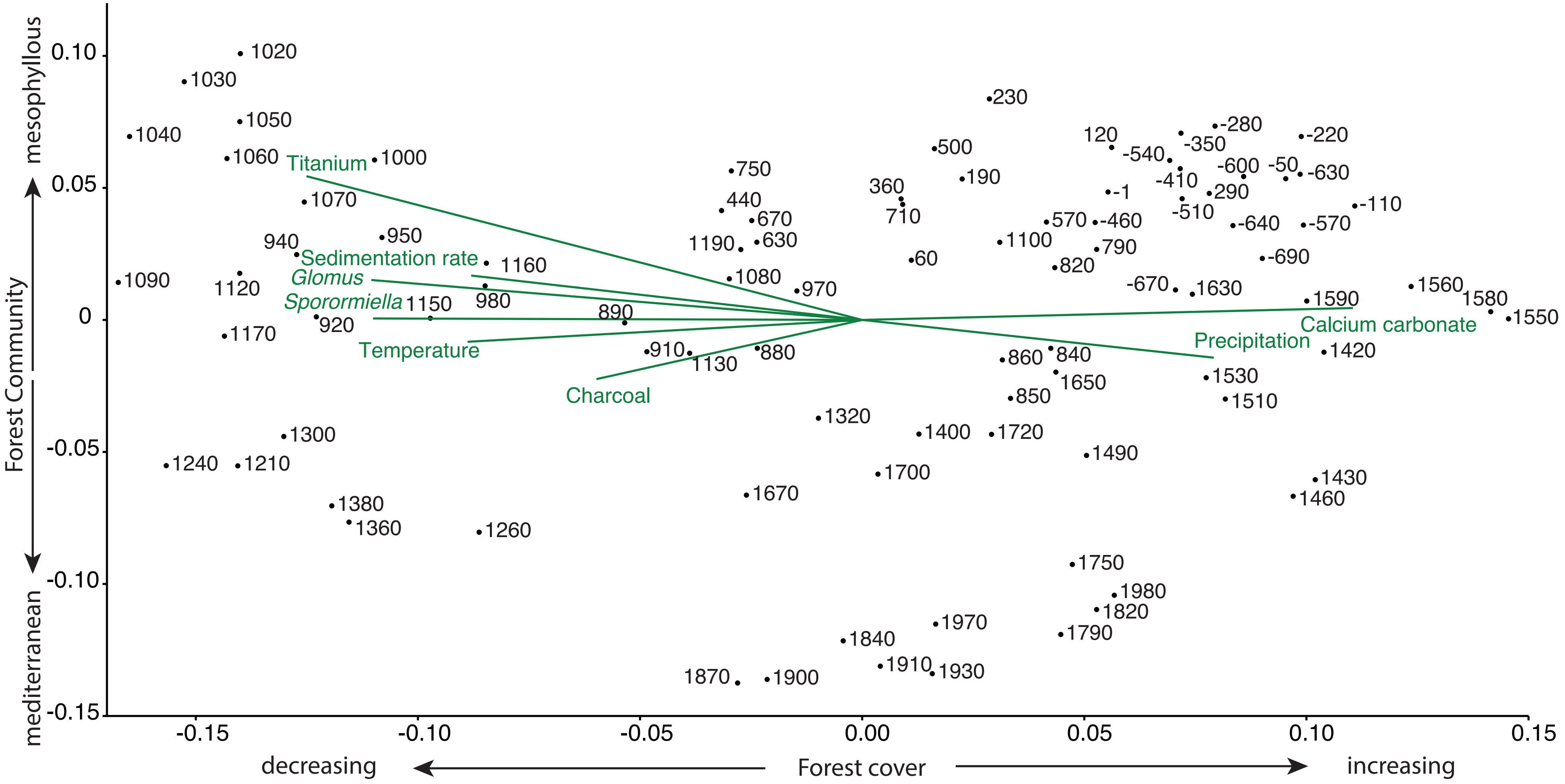

Fig. S1. Non-metric multidimensional scaling based on Bray-Curtis similarity index (stress = 0.13; R2 of the axis 1: 0.64 and of axis2: 0.19) of the plant taxa from Lago Lungo. Vectors show the correlation with the environmental variables other than pollen; non-pollen palynomorphs, charcoal, climate proxies, sedimentation and geochemical proxies (green vectors and green text). Vector length is arbitrarily scaled to make a readable biplot; only their directions and relative lengths should be considered; associated numbers represent ages (year BCE/CE) for each pollen strata.

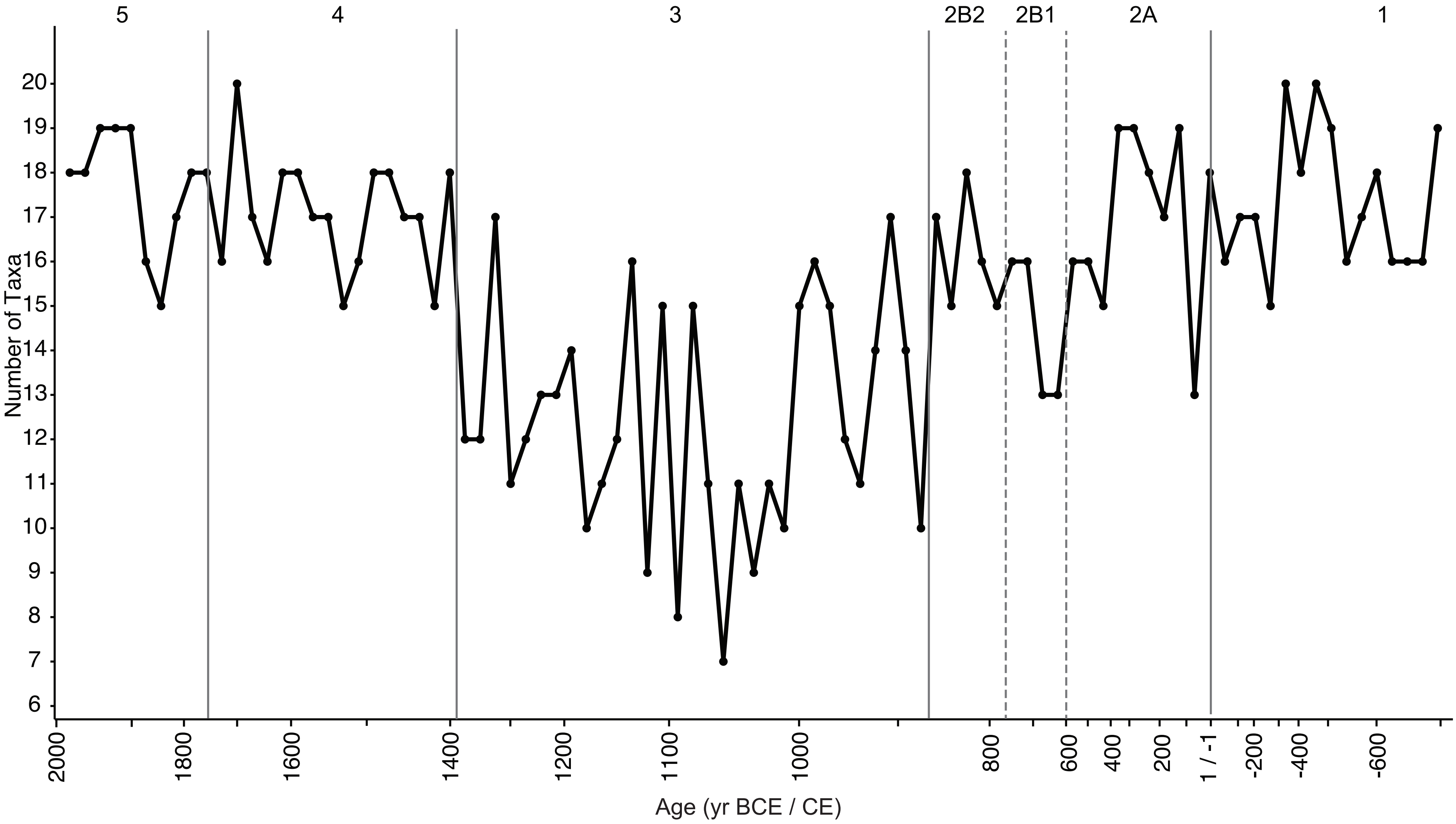

Fig. S2. Temporal trends in the richness of woody taxa.
